# Interventions to support the psychological empowerment of nurses: a scoping review

**DOI:** 10.3389/fpubh.2024.1427234

**Published:** 2024-12-09

**Authors:** Liebin Huang, Ming Liu, Xin Wang, Meihua Hsu

**Affiliations:** ^1^Faculty of Health Sciences and Sports, Macao Polytechnic University, Macao, Macao SAR, China; ^2^Peking University Health Science Center – Macao Polytechnic University Nursing Academy, Macao Polytechnic University, Macao, Macao SAR, China

**Keywords:** nurses, empowerment, psychological empowerment, psychological interventions, scoping review

## Abstract

**Background:**

Establishing an empowering work environment is significantly contributing to nurse’s job satisfaction, performance, retention, and organizational success. This study aimed to conduct a scoping review to chart and synthesize current research on interventions to support nurses’ psychological empowerment.

**Methods:**

Ten databases were searched, including PubMed/Medline, Web of Science, Scopus, Embase, EBSCOhost, Cochrane Library, CNKI, Wanfang, VIP, and OpenGrey, following the Joanna Briggs Institute’s methodology for scoping reviews. The search encompassed literature from its inception to 5 September 2024. The selection of studies followed predetermined inclusion and exclusion criteria. A manualized systematic quality assessment method was applied to the included studies, and the extracted data were charted using a series of tables.

**Results:**

Eleven studies were included. Seven studies reported the theoretical framework used. The interventions are all educational and are divided into two main sections: theoretical learning and applied practice. The duration of the interventions spanned a wide range of hours. The intervention format was based on offline training. The participants included both nurse managers and nurses. The evaluation measures were mostly multiple time points using the Spreitzer Psychological Empowerment Instrument. The interventions were generally effective, although some studies reported different results.

**Conclusion:**

Research on psychological empowerment interventions for nurses is still in the developmental phase, with preliminary evidence validating their positive effects. Future research should focus on conducting randomized controlled studies with larger sample sizes, selecting appropriate theoretical frameworks to design interventions, enriching the content and form of interventions, and strengthening evaluation measures to improve the quality of psychological empowerment interventions for nurses.

**Systematic review registration:**

OSF, https://doi.org/10.17605/OSF.IO/W7ZG6.

## 1 Introduction

Under the situation of a global shortage of nursing workforce and stressful working conditions, it is crucially important to create a favorable working environment to stabilize the nursing workforce and to enhance the nurses’ working engagement and morale (1). A positive work environment encompasses effective leadership, harmonious collegial relationships, and ample organizational support and resources, among other factors (2). This work environment is often referred to as an empowered work environment (3, 4). Nurses who experience a sense of empowerment have been shown to perceive lower job stress, higher job satisfaction, and higher willingness to retain their jobs (5). Chandler first proposed the process of empowerment in nursing (6). It covers two aspects: structural empowerment proposed by Kanter (7), and psychological empowerment proposed by Spreitzer (8).

Structural empowerment theory suggests that when employees have access to empowering conditions, including information, resources, support, and professional development opportunities, their performance improves (7). Psychological empowerment stresses the individual’s perception of empowerment. Spreitzer defines psychological empowerment as an employee’s psychological perception or attitude toward their work and organizational role (8). It represents intrinsic task motivation and reflects an individual’s desire and ability to influence their work and workplace (8).

Psychological empowerment comprises four cognitive dimensions: (1) meaning, or the value of the work goal about one’s ideals and standards; (2) competence, or one’s work role efficacy; (3) self-determination, or an individual’s sense of autonomy; and (4) impact, or one’s belief to make a difference or produce the intended effect in one’s task context (9). These components enable employees to align their work with their values, beliefs, and behaviors, meet job demands, and influence organizational decisions (10). Nurses’ psychological empowerment is defined as the nurses’ perceptions of meaningful nursing work, ability to perform professional tasks, involvement in decision-making within the organization, and professional efficiency (3, 11). Research has shown that empowered nurses are more likely to have positive beliefs about their ability to make meaningful contributions to the workplace than nurses who are not psychologically empowered, and these positive beliefs, therefore, enhance job satisfaction (12).

Psychological empowerment as an internal motivational force is important for nurse outcomes. Studies have shown that high levels of psychological empowerment are significantly associated with reductions in stress, burnout, and intention to leave, as well as increased organizational commitment and innovative behavior (12–15). In addition, the psychological empowerment of nurses is important for patient outcomes and has been described as a key factor in improving quality of care and patient safety (12, 16). Empowered nurses exhibit higher levels of confidence in their capacity to deliver quality care to patients (17). The nurse/patient empowerment model suggests that nurses with higher levels of psychological empowerment are more likely to collaborate with patients, which encourages patient empowerment behaviors that lead to better patient functioning and improved health outcomes (18).

Therefore, enhancing nurses’ psychological empowerment has emerged as a prominent focus across all levels of healthcare management, leading to a gradual rise in intervention studies aimed at strengthening nurses’ psychological empowerment. Organizational leadership stands out as a significant factor in this realm. The efficacy of organizational leadership directly impacts employees. Research has underscored the direct and positive influence of virtuous leadership styles like transformational (19), authentic and ethical (20), servant (21), and humble leadership (22) on nurses’ psychological empowerment. In this context, nursing managers can empower their team members psychologically by demonstrating appropriate behaviors. Leadership styles and skills are teachable and can be cultivated, particularly when training programs are complemented by institutional support processes (23). Nursing managers who implement empowerment strategies gleaned from these programs can effectively empower their followers through improved interactions (24). Current research is primarily focused on intervention studies that center on leadership development to promote the psychological empowerment of nurses (25). For instance, a study in Turkey devised a comprehensive educational program rooted in authentic leadership to offer training for nurse leaders, intending to assess the efficacy of nurse leaders and their followers in terms of psychological empowerment (24).

However, as psychological empowerment is essentially an internal experience, recognized as a positive psychological phenomenon (26), associated with the nurse’s self-perception, interventions aimed at altering the work environment might adversely affect nurses’ adaptability to change, potentially increasing work-related stress (27). Therefore, some studies focused on providing direct psychological support to nurses to empower them, emphasizing their psychological well-being (28, 29). The types and contexts of these support interventions vary widely. Coupled with factors shaped by different theoretical frameworks, the specifics of the interventions, and outcome indicators used contribute to notable differences in the results of these studies, resulting in a high degree of heterogeneity. To address this knowledge gap and comprehensively explore new evidence, a scoping review represents an appropriate approach. By employing standardized and systematic literature searches and screening, a scoping review can elucidate the depth and breadth of existing research, summarize findings or identify research gaps, and provide a higher level of evidence compared to traditional reviews (30). Therefore, this study aimed to conduct a scoping review to chart and synthesize the evidence available in the literature regarding interventions aimed at supporting the psychological empowerment of nurses, while also identifying any existing gaps, to inform future research on the psychological empowerment of nursing staff.

## 2 Methods

### 2.1 Design

A scoping review was conducted for this study using the Joanna Briggs Institute (JBI) methodology (31). The seven steps were followed: (1) identifying the research questions, (2) clarifying the inclusion and exclusion criteria, (3) using the systematic search strategy, (4) study selection, (5) data extraction and graphing, (6) data analysis, and (7) presentation of results (32). A protocol for this scoping review was published in the Open Science Framework registries on 26 April 2024. In addition, the PRISMA-ScR checklist was used to ensure the quality of this scoping review (33).

### 2.2 Identifying the research questions

The research questions were established based on the JBI’s principles of population-concept-context (PCC) (32), where the population (P) is registered nurses, the concept (C) is interventions to improve the level of psychological empowerment of nurses, and the context (C) is research conducted in the work environment. Based on the above principles, the final research question for this study was determined as follows: What interventions have been identified in existing research to augment the level of psychological empowerment of nurses within the work environment?

### 2.3 Clarifying the inclusion and exclusion criteria

Inclusion criteria: (1) Articles published in English or Chinese with full-text access; (2) The study population was nurses, regardless of their workplace (such as inpatient wards, community settings, long-term care facilities, psychiatric hospitals, and so on); (3) The studies included research related to interventions to improve nurses’ psychological empowerment; (4) Only original studies with intervention study designs will be considered, including randomized controlled study designs, before-and-after controlled studies, and non-randomized controlled studies.

Exclusion criteria: (1) Inconsistency with the research question; (2) Literature type of conference abstracts, news reports, letters, and books; (3) Repeated publication or secondary analysis of the same study.

### 2.4 Search strategy

Six English databases (PubMed/Medline, Web of Science, Scopus, Embase, EBSCOhost, Cochrane Library), three Chinese databases (China National Knowledge Infrastructure, CNKI), Wanfang, VIP, and the grey literature database OpenGrey^1^ were searched using subject terms combined with free terms. The search timeframe for the database was from its establishment until 5 September 2024. The English search terms included nurs*, personnel, nursing, nursing personnel, registered nurses, nurse, registered, nurses, registered, registered nurse, nurse practitioner*, staff nurse*, nursing staff*, nurse clinician*, licensed practical nurse*, nurse midwife*, psychological empowerment, psychological-empowerment. The Chinese search terms included (心理授权or护士or护理人员or 临床护士or随机对照试验or随机or 对照or RCT or干预前后or干预or前后or 试验). The full search strategy is provided in Supplementary material.

### 2.5 Study selection and data extraction

EndNote X9 software was used to import the literature and remove duplicates. In the first step, two researchers who had received specific training independently reviewed the titles and abstracts of the literature, following the inclusion and exclusion criteria and the study’s purpose, for initial screening. In the second step, the full text of the literature was thoroughly examined for further screening. If any discrepancies arise between the two steps mentioned above, a third researcher assisted in reaching a judgment. Extracted data included authors, publication date, country, study design and sample size, participant characteristics, intervention characteristics, evaluation measures, and study findings.

### 2.6 Quality appraisal

The Template for Intervention Description and Replication (TIDieR) checklist and guidelines developed by Hoffmann et al. (34) were used to evaluate the quality of the studies included in the review. Each article was evaluated against 12 criteria to identify the strengths and weaknesses of intervention studies. A percentage score was computed to measure how well each paper met the quality assessment criteria. Two researchers independently carried out this evaluation, deliberated on the findings, and reached a consensus on the final quality assessment score for each paper indicated in Table 1.

**Table 1:** Quality assessment table.

Intervention studies	01	02	03	04	05	06	07	08	09	10	11
1. Brief name: Provide the name or a phrase that describes the intervention?	√	√	√	√	√	√	√	√	√	√	√
2. Why: Describe any rationale, theory, or goal of the elements essential to the intervention?	√	√	√	√	√	√	√	√	√	√	√
3. What (materials): Describe any physical or informational materials used in the intervention, including those provided to participants or used in intervention delivery or in training of intervention providers.	√	√	√	√	√	√	√	√	√	√	√
4. What (procedures): Describe each of the procedures, activities, and/or processes used in the intervention, including any enabling or support activities?	√	√	√	√	√	√	√	√	√	√	√
5. Who provided: For each category of intervention provider (for example, psychologist, nursing assistant), describe their expertise, background and any specific training given?	√	×	√	×	×	×	×	×	√	√	√
6. How: Describe the modes of delivery (such as face to face or by some other mechanism, such as internet or telephone) of the intervention and whether it was provided individually or in a group?	√	√	√	√	√	√	√	√	√	√	√
7. Where: Describe the type(s) of location(s) where the intervention occurred, including any necessary infrastructure or relevant features?	√	√	√	√	√	√	√	√	√	√	√
8. When and how much: Describe the number of times the intervention was delivered and over what period of time including the number of sessions, their schedule, and their duration, intensity or dose?	√	√	√	√	√	√	√	√	√	√	√
9. Tailoring: If the intervention was planned to be personalized, titrated or adapted, then describe what, why, when, and how?	×	×	√	√	×	√	×	×	√	×	×
10. Modifications: If the intervention was modified during the course of the study, describe the changes (what, why, when, and how)?	×	×	×	×	×	×	×	×	×	×	×
11. How well (planned): If intervention adherence or fidelity was assessed, describe how and by whom, and if any strategies were used to maintain or improve fidelity, describe them?	√	√	√	√	√	√	√	√	√	√	√
12. How well (actual): If intervention adherence or fidelity was assessed, describe the extent to which the intervention was delivered as planned?	√	√	√	√	√	√	√	√	√	√	√
Quality score (%)	83.3	75.0	91.6	83.3	75.0	83.3	75.0	75.0	91.6	83.3	83.3

### 2.7 Data analysis and presentation of results

For the extracted data, a narrative summary and tables were used to describe the characteristics of the included studies after a uniform re-review by all researchers, and the results were described using descriptive analysis and basic content analysis.

## 3 Results

### 3.1 Search results

**Figure 1:**
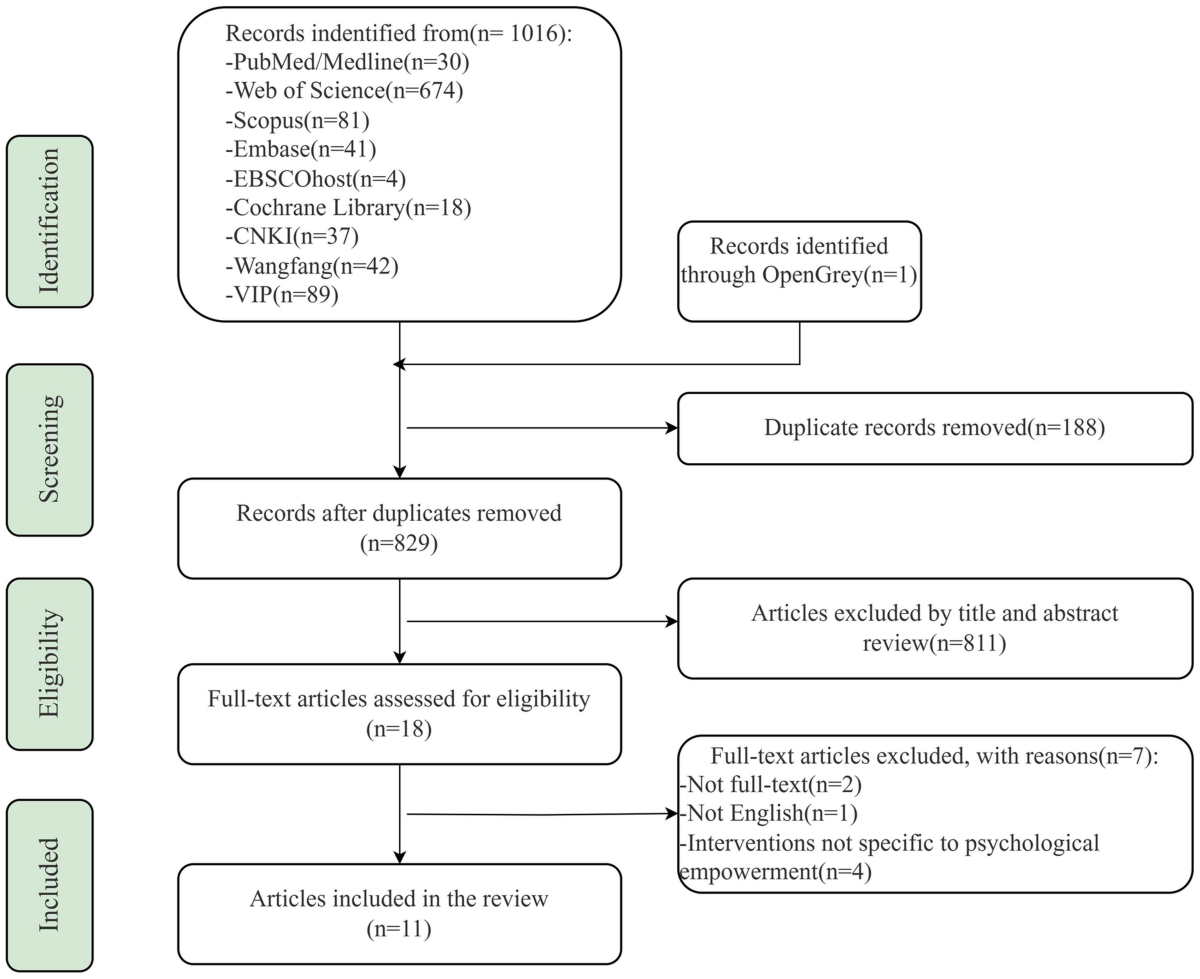
Literature screening process and results.

A total of 1,017 articles were retrieved from ten databases, of which one was from the grey literature database. Following the de-duplication process and the exclusion of clearly irrelevant articles, 18 full-text articles were obtained, and finally, 11 were included in this study. The specific literature screening process is shown in the PRISMA flowchart (Figure 1). Quality assessment scores for the included studies ranged from 75 to 91.6% based on the guidelines put forth by Hoffmann et al. (34).

### 3.2 Study characteristics

Table 2 summarizes the study characteristics. In terms of study design, most studies used a one-group pretest-posttest design (*n* = 6) (24, 29, 35, 36) and a pretest-posttest design with a non-equivalent control group (*n* = 4) (27, 37–39), while others included 1:1 parallel randomized controlled trials (*n* = 1) (40). A total of six countries were included, with the most studies published in Turkey (*n* = 3) (24, 27, 36) and China (*n* = 3) (28, 37, 41), while other countries including the USA (*n* = 2) (21, 27), Sweden (*n* = 1) (38), Canada (*n* = 1) (23), and Finland (*n* = 1) (35). Intervention occurrence was from the summer of 2005 to May 2022. Sample sizes included in the studies ranged from 16–212 for a total sample size of 950, and sample sources included nurses (*n* = 5) (27, 28, 37, 38, 41), nurse managers (*n* = 4) (23, 29, 35, 40), and nurses and nurse managers (*n* = 2) (24, 36). The majority of these studies were multicenter (*n* = 6) (23, 29, 35, 37, 38, 40) and the remaining studies (*n* = 5) were single-center (24, 28, 29, 36, 41).

**Table 2:** Characteristics of included studies.

Reference	Study design	Country	Intervention period	Participant number/type	Single/Multiple center
Chang et al. (37)	Non-equivalent control-group and pretest-posttest study	China-Taiwan	summer 2005	61 (public health nurses)	Multiple center
Dirik and Seren Intepeler (24)	One-group pretest–posttest design	Turkey	2018.12–2020.1	189 (36 head nurses and 153 nurses)	Single center
Engström et al. (38)	Pre-test–post-test design with an intervention and comparison group	Sweden	2006–2007 (7 months)	46 (nursing home for the older adult and home care service nurses)	Multiple center
MacPhee et al. (23)	Pre-test–post-test design with an intervention and comparison group	Canada	2007–2010 (selected nurse leaders trained during the period)	128 (nurse leaders with less than 3 years experience)	Multiple center
Sawyer et al. (29)	One-group pretest-posttest design (mixed methods study)	USA	2021.5–2021.7	16 (nurse manager)	Single center
Sawyer et al. (40)	1:1 parallel randomized controlled trial	USA	2022.3–2022.5	77 (30 nurse managers and 47 assistant nurse managers)	Multiple center
Terkamo-Moisio et al. (35)	One-group pretest–posttest design	Finland	2019.8–2021.3	25 (current or prospective nurse leaders)	Multiple center
Yilmaz and Duygulu (36)	A pre-experimental, one-group, pretest-posttest design	Turkey	2018.4–2018.11	212 (38 unit charge nurses and 174 staff nurses)	Single center
Özbaş and Tel (27)	Pretest-posttest design with an intervention and comparison group	Turkey	Not mentioned	82 (nurses who work on adult inpatient oncology clinics)	Multiple center
Xiong et al. (28)	One-group pretest–posttest design	Chinese Mainland	2018.1–2018.3	40 (nurse staff)	Single center
Cheng et al. (41)	One-group pretest–posttest design	Chinese Mainland	74 (male nurse)	74 (male nurse)	Single center

### 3.3 Theoretical framework

Table 3 summarizes the theoretical framework of the intervention. Among the 11 included studies, theoretical frameworks were reported in seven of them. Two studies utilized theoretical frameworks based on psychological empowerment theory: the cognitive empowerment model (37) and workplace empowerment theory (39). The cognitive empowerment model suggests that environmental events, such as input from managers, charismatic attraction, training sessions, and mentoring advice, directly impact an individual’s task assessment, which includes dimensions like meaning, competence, impact, and self-determination. Successful task assessment leads to higher levels of empowered behavioral outcomes (9). The workplace empowerment theory emphasizes the process of structural empowerment (organizational support) leading to psychological empowerment, which, in turn, influences various outcomes like job satisfaction, organizational commitment, and innovativeness (39). One of the remaining five studies employed psychodrama theory, which aligns closely with Spreitzer’s definition of psychological empowerment theory (27) and emphasizes the need for an external perspective to redefine one’s self. Two studies used group psychological counseling theory, a psychological intervention form offering guidance and support within a team setting (42). The other two studies were different study designs of the same intervention program, using the theoretical frameworks of Mindfulness, Acceptance and Commitment Therapy (ACT), and Cognitive Behavioral Therapy (CBT), as well as Post-Traumatic Growth Theory, which is a common theory in psychotherapy.

**Table 3:** Theoretical framework.

Reference	Theoretical framework utilized (if applicable/reported)
Chang et al. (37)	Thomas&Velthouse’s cognitive empowerment model and the reflective empowerment education approach based on Paulo Freire
Dirik and Seren Intepeler (24)	Not reported
Engström et al. (38)	Not reported (mentioned the theoretical basis, but no specific theoretical framework)
MacPhee et al. (23)	workplace empowerment theory
Sawyer et al. (29)	Mindfulness, Acceptance, and Commitment Therapy (ACT), and Cognitive Behavioral Therapy (CBT); Post-Traumatic Growth theory
Sawyer et al. (40)	Mindfulness, Acceptance, and Commitment Therapy (ACT), and Cognitive Behavioral Therapy (CBT); Post-Traumatic Growth theory
Terkamo-Moisio et al. (35)	Not reported
Yilmaz and Duygulu (36)	Not reported
Özbaş and Tel (27)	Psychodrama theory
Xiong et al. (28)	Group psychological counseling theory
Cheng et al. (41)	Group psychological counseling theory

### 3.4 Intervention description, evaluation measures, and study findings

Table 4 summarizes the intervention descriptions, evaluation measures, and study findings. The interventions are all educational. Regarding the content of the interventions, most studies were divided into two parts: theoretical learning and applied practice. The theoretical learning encompassed the following: ① Training on the theme of psychological empowerment, elucidating the concept, connotation, and related theoretical knowledge of psychological empowerment; ② Training on the theme of the work environment, exploring the influence of the work environment on psychological empowerment, strategies for improving the work environment, and methods for exerting psychological empowerment in different work environments; ③ Education on the theme of self-awareness, introducing nurses’ perceptions of their roles, fostering self-efficacy, and ways to enhance psychological empowerment through self-awareness, and ④ Teamwork-themed education, through the organization of teamwork group discussions, prompts nurses to share their experiences of teamwork and guides them to learn how to play the role of psychological empowerment in the team. Four studies clearly emphasized the importance of applying the training content to the work environment (24, 36, 37, 39), such as through the use of learning materials, and action plans, which allowed participants to apply the reflections and actions learned in the classroom and seminars to their actual work environment. Additionally, five out of the remaining seven studies proposed enhancing the training effect through face-to-face workshops (*n* = 1) (35) or feedback learning (*n* = 4) (27, 29, 40, 41). The frequency and duration of theoretical training varied depending on the intervention program’s content, spanning from a frequency of up to nine times a week to eight times in nine months, with the shortest duration being 12 h and the longest lasting up to 19 months. The length of practice also varies, ranging from a few group workshops for the shortest to a year for the longest. Most of the training was conducted offline (*n* = 9) (24, 27–29, 36–38, 40, 41). Offline training typically involved psychological empowerment courses, group meetings, group workshops, group counseling, individual exercises, improvisation, reflection and experiential learning, exercise books, and written notes. Two studies utilized blended training approaches. One study initially conducted a four-day offline study, followed by online knowledge network support for trainees (39). Another study employed a 19-month online program, followed by face-to-face offline workshops (35). Regarding the participants in the training, the majority were care managers (*n* = 5) (23, 24, 29, 35, 40). The remaining studies involved nurses (*n* = 3) (27, 37, 38) or both nurses and care managers (*n* = 1) (36). It is worth noting that this distribution does not align with the source of the sample. In the study by Dirik and Seren Intepeler (24), the training primarily targeted care managers, measuring pre and post-changes in their psychological empowerment. However, data related to the nurses’ follower role were also collected in that study.

**Table 4:** Intervention description, evaluation measures, and study findings.

Reference	Intervention description	Evaluation measures	Study findings
Chang et al. (37)	*Empowerment-based educational program* The authors and two trained lead public health nurses (LPHNs) facilitated a four-week empowerment education program specifically designed for PHNs. The program was conducted once a week for six hours and comprised empowerment classes and group workshops, totaling 24 h of engagement. Additionally, participants were encouraged to apply their newly acquired knowledge to real work situations, fostering feedback exchange and experience sharing.	Organizational empowerment scale, Spreitzer Psychological Empowerment Instrument (PEI), Job Satisfaction Scale, Job Productivity Scale, and Innovative Behavior Scale. They were administered 4 weeks before and after testing in the intervention and control groups.	After 4 weeks, the experimental group reported significantly higher overall levels of psychological empowerment, competence, and impact dimensions compared to the control group.
Dirik and Seren Intepeler (24)	*Multi-faceted educational program* The program consisted of 10 major training sessions held in the hospital training room, along with three additional intensive training sessions in various units. The main training course spanned 3 months and involved nurse leaders, with each session lasting approximately 45 min. The training covered fundamental knowledge and skills in authentic leadership, empowerment, and patient safety. To enhance learning effectiveness, the format included group work, question-answer sessions, and case studies. Intensive training took place six months after the completion of the main course, addressing the specific learning needs of nurse leaders in each unit. These sessions lasted around 60 min on three occasions and provided supplementary support based on their current learning status. To reinforce learning, participants were provided with workbooks and keychains at the end of the course.	Safety Climate Survey (SCS), Authentic Leadership Questionnaire (ALQ), Conditions of Work Effectiveness Questionnaire-II (CWEQ-II), and Spreitzer Psychological Empowerment Instrument (PEI). Measurements were taken separately for nurse leaders and nurses both before and after the intervention. For nurse leaders, measurements were taken as a follow-up before the main training course, immediately after the main training course, and six months after the main training course (a total of three measurements). For nurses, measurements were taken as a follow-up after the nurse leader’s main training course and six months after the main training course (a total of two measurements).	After the intervention, the meaning score for psychological empowerment among nurse leaders was found to be ≥4, with no significant difference between the mean scores (*p* = 0.062). However, when examining the subdimensions, a significant difference was observed in the ‘impact’ subdimension (*p* = 0.041). In terms of nurse followers, the meaning score for psychological empowerment increased from 3.96 to 4.10. A related samples *t*-test indicated a significant difference between the two measures (*p* = 0.013). Similarly, significant differences were found within the subdimension of ‘influence’ (*p* = 0.004).
Engström et al. (38)	The intervention/training program comprised eight group sessions, with each session lasting approximately 1.5 h. The sessions were conducted over nine months. The training target is nursing home nurses. Each group session had a designated facilitator, who was a registered nurse, a sociologist, and a social worker, respectively.	Spreitzer Psychological Empowerment Instrument (PEI), Psychosocial aspects of job satisfaction scale. Measured before and after the intervention.	At post-test, scores for the factors self-determination (intervention group means 5.4, SD 1.0; comparison group means 4.7, SD 1.2, *p* = 0.039) and impact (intervention group means 5.6, SD 0.8; comparison group means 4.7, SD 1.2, *p* = 0.015) and the total scale of empowerment (intervention group means 5.9, SD 0.6; comparison group means 5.3, SD 0.8, *p* = 0.011) were significantly higher in the intervention group than in the comparison group. When compared over time within the respective groups, the results showed no statistically significant differences in empowerment for either the intervention group or the control group.
MacPhee et al. (23)	*Nursing Leadership Institute (NLI) program* There are two components to the program. Participants are nurse leaders with less than 3 years of experience. The first component is a 4-day offline residential workshop that focuses on instructional leadership content and includes interactive learning sessions. The second component is a 1-year practice that involves an online knowledge network designed to provide support to nursing leaders.	Conditions of Work Effectiveness (II) Scale (CWEQII), Spreitzer Psychological Empowerment Instrument (PEI), and Leader Empowering Behaviors Scale (LEBS). The testing was conducted one year before and after the intervention in both the intervention and control groups. In the intervention group, the first measurement was taken during the NLI workshop, and the second measurement was taken one year after the workshop.	The mean overall level of psychological empowerment in the intervention group increased from 3.89 to 4.05 after one year. A relevant samples *t*-test revealed a significant difference with *t* = 3.31, *p* < 0.01. However, no statistical significance was found in the control group.
Sawyer et al. (29)	*Psychoeducational group program* The program combines education, therapeutic process, skill development, and social support in nine weekly 90-min sessions. The course incorporates three main approaches: education, self-reflection (providing workbooks for note-taking), and experiential learning. The participants in this program were nurse managers.	Posttraumatic Growth Inventory Scale, Brief Resilience Scale, Self-Reflection and Insight Scale, Self-Compassion Scale, Spreitzer Psychological Empowerment Instrument (PEI), Perceived Stress Scale, Maslach Burnout Inventory Scale, Brief Index of Affective Job Satisfaction. Measured before and after the intervention.	The overall mean score on the Psychological Empowerment Instrument was significantly higher at follow-up than at baseline (5.81 vs. 6.06, *t* = −2.50, *p* = 0.03). Meaning scores in the following domains were significantly higher after the intervention: Competence (5.42 vs. 5.96, *t* = −3.81, *p* < 0 0.01) and Impact (5.90 vs. 6.19, *t* = −2.21, *p* = 0 0.04). There were no statistically significant differences in the mean scores in the domains of Meaning (6.42 vs. 6.38 vs. 6.23) and Self-Determination (5.52 vs. 5.73 vs. 5.69).
Sawyer et al. (40)	*Psychoeducational group program* The program consists of nine 90-min group sessions held once a week for nine weeks. The sessions include an introduction session, two resilience sessions, two insight sessions, one self-compassion session, two empowerment sessions, and a closing session. Mindfulness exercises underpinned the learning and were used throughout each session. The participants in this program are nurse managers.	Posttraumatic Growth Inventory Scale, Brief Resilience Scale, Self-Reflection and Insight Scale, Self-Compassion Scale, Spreitzer Psychological Empowerment Instrument (PEI), Perceived Stress Scale, Maslach Burnout Inventory Scale, Brief Index of Affective Job Satisfaction. Five measurement times were taken: pre-intervention, 1-month post-intervention follow-up, 3-month follow-up, 6-month follow-up, and endpoint.	Among intervention group participants, the overall mean score on the PEI was significantly higher at the endpoint than at baseline (5.41 vs. 5.82, *t* = −2.86, *p* = 0.007). Meaning scores in three subdomains were significantly higher after the intervention: Meaning (5.82 vs. 6.20, *t* = −2.37, *p* = 0.024), Competence (5.35 vs. 5.97, *t* = −4.90, *p* < 0.001), and self-determination (5.30 vs. 5.63, *t* = −2.08, *p* = 0.045). There was no significant difference among intervention group participants in the meaning score in the domain of Impact (5.22 vs. 5.49).
Terkamo-Moisio et al. (35)	*Continuing Nursing Leadership Education program* The program, delivered by a partnership of five universities, lasts for 19 months and includes seven courses, including leadership theory. The courses encompass various learning methods, including web-based learning through Moodle, virtual environments, and face-to-face workshops. The program is designed for nursing leaders.	Conditions of Work Effectiveness Questionnaire (CWEQ-II), Work Empowerment Questionnaire. Three data collection time points were included: the first at the start of CLEP from October 2019 to February 2020, the second at the end of CLEP (first follow-up) during March and April 2021, and the third 8 months after the end of CLEP (second follow-up) from November 2021 to January 2022.	As a result of the intervention, participants’ levels of total psychological empowerment were enhanced.
Yilmaz and Duygulu (36)	*Education-based empowerment program* The program comprises a total of 34 h, which includes 14 h of theoretical training divided into 7 sessions of 2 h each. Additionally, there are 12 h of group work distributed across 9 sessions of 1 to 1.5 h each. Furthermore, the program includes 8 h of individual work, consisting of 6 sessions of 1 to 1.5 h each. The participants in this program were unit charge nurses and staff nurses.	Spreitzer Psychological Empowerment Instrument (PEI), Hospital Survey on Patient Safety Culture Scale. Data was collected before and after the intervention, as well as for 4 months afterward.	As a result of the intervention, there was a statistically significant increase in the scores on the Psychological Empowerment Scale, specifically on the Competence and Meaning subscales.
Özbaş and Tel (27)	*Psychological empowerment program based on psychodrama* The training is facilitated by a psychodrama writer and is specifically designed for oncology nurses. The training sessions are conducted once a week, with each session lasting 2 h, and the entire program spans over 10 weeks. The content of the training covers various topics such as group contracting, coping with stress and cognitive distortions, relaxation techniques, problem-solving, self-awareness, empathy, dispute resolution, assertiveness training, and the theme of death. During the training, participants engage in learning through improvisation by setting up specific scenario modules during each session. After each session, participants were allowed to provide feedback, and their awareness of various aspects including communication style, patterns, emotions, thoughts, and behaviors related to the discussed topics by both themselves and other group members was recorded.	Spreitzer Psychological Empowerment Instrument (PEI), Nurse Work Empowerment Scale (NWES), and Maslach’s Burnout Inventory (MBI). Measurements were taken before the intervention, 1 month, and 3 months after the intervention.	The total psychological empowerment scores of nurses in the study group significantly increased after they participated in a psychodrama-based psychological empowerment program.
Xiong et al. (28)	*Group psychological counseling method* This method includes theoretical teaching and group psychological counseling. For theoretical teaching, psychological experts are invited to teach psychological theories, covering aspects such as stress and health, doctor-patient relationships, etc. This is carried out once every three weeks for a total of two times. For group psychological counseling, psychotherapists are invited to conduct counseling for nurses by using the theories and techniques of group psychological counseling. The theme of the counseling program is “Happy Work,” which contains 6 units. It is conducted once a week, each time for 3 h, and the intervention lasts for 6 weeks.	The Chinese version of Spreitzer’s Psychological Empowerment Instrument (PEI). Data were collected before and one week after the intervention.	After the intervention, the nurses’ scores on impact, competence, self-determination, meaning, and the total score on the psychological empowerment scale were all significantly higher than those before the intervention.
Cheng et al. (41)	*Group Psychological Intervention Program* The program integrates theoretical training and behavioral training activities. It involves a 5-week team-based psychological intervention, randomly dividing participating male nurses into 5 groups. The intervention occurs 1 or 2 times per week, each lasting 3–4 h. The theme of the intervention is ‘Know Yourself, Be Happy at Work’. Each session has an activity theme, covering topics such as understanding nursing, working together, stress management, emotional control, and thinking about the future. In addition, group members organize regular meetings once a week throughout the process to share their experiences to enhance the intervention’s effectiveness.	The Chinese version of Spreitzer’s Psychological Empowerment Instrument (PEI), The Chinese version of the Nurses’ Professional Identity Scale. Data were collected before and two weeks after the intervention.	The nurses’ scores on impact, competence, self – determination, meaning, and the total score on the psychological empowerment scale after the intervention were all significantly higher compared with those before the intervention.

Regarding evaluation measures, nurses’ psychological empowerment was measured at baseline and at different time points using various instruments depending on the study. The most commonly used instrument was the Spreitzer Psychological Empowerment Instrument (PEI), which was utilized in ten (23, 24, 27–29, 36–38, 40, 41) out of the eleven included studies. This instrument comprises four sub-dimensions: meaning, competence, self-determination, and impact (8). It is considered the most widely used measure of psychological empowerment in nursing (12). One study (35) used the Work Empowerment Questionnaire, which is divided into three sections: verbal, behavioral, and outcome empowerment (43). In addition, job satisfaction (29, 37, 38, 40) often appeared together as measures, and the remaining measures included structural empowerment, work efficiency, innovative behavior, safety climate, authentic leadership, and so on. The timing of the measurements varied across the studies. They could be broadly categorized into pre- and post-intervention measurements (*n* = 6) (23, 28, 29, 37, 38, 41) and pre- and post-intervention with multiple measurements at different time stages during follow-up (*n* = 5) (24, 27, 35, 36, 40). For example, one study measured baseline data for nurse leaders, followed by data collection after the first phase of the training course, followed by another six-month interval (24). Another study measured baseline data for both intervention and control groups, followed by measurements at one month, three months, six months, and endpoint nodes, resulting in a total of five measurement points (40). Other studies conducted measurements at baseline, immediately after the intervention, and at various follow-up time points (e.g., four months, eight months) to assess the intervention’s impact (27, 35, 36).

Regarding the outcomes, the majority of studies indicated that interventions had a significant positive impact on the overall levels of psychological empowerment among nurses (*n* = 9) (23, 24, 27–29, 35, 37, 40, 41). Particularly noteworthy were the significant improvements observed in the impact subdimension (*n* = 5) (24, 28, 29, 40, 41) and the competence subdimension (*n* = 4) (28, 29, 40, 41). In two of the remaining studies, one reported enhancements specifically in the competence and meaning subdimensions, although the overall level of improvement was not statistically significant (36). It is important to mention that Maria Engström (38) found significantly higher results in the intervention group compared to the control group in terms of self-determination, impact, and overall level demonstrated no statistically significant differences comparing the groups. This suggests that further investigation is needed to determine the true efficacy of their intervention.

## 4 Discussion

This scoping review collated evidence from multiple countries, reflecting the global concerns about the psychological empowerment of nurses.

Among the available study designs, the use of non-equivalent control group pretests and posttests, as well as randomized controlled trials, is commendable as they provide better opportunities to establish causal relationships. Additionally, most of the studies employed multicenter designs. For instance, the study conducted in Taiwan, China involved participants from two health bureaux (37), and the Swedish study recruited participants from a nursing home and a home care service (38). However, it is crucial to note that these multicenter studies still possess certain limitations. Many of these multicenter studies had small sample sizes, and the use of convenience sampling methods did not allow for effective control of confounding factors. The small and homogenous samples limited the ability to conduct detailed analyses, undermining the intervention’s impact in the current study. This limitation indicates the need for future research to incorporate larger sample sizes and randomized controlled studies to enhance the effectiveness and generalizability of psychological empowerment interventions.

The majority of the included studies in this review provided descriptions of the theoretical frameworks utilized in the intervention programs. However, four studies did not report on the theoretical frameworks employed. The absence of such reporting poses challenges in fully comprehending how interventions can effectively support nurses’ psychological empowerment. Future studies should prioritize the application of theoretical frameworks to more effectively guide and explain how the interventions impact nurses’ psychological empowerment. Choosing the right theoretical framework for an intervention is essential; therefore, programs aimed at fostering psychological empowerment should utilize theories that align with their foundational models.

The psychological empowerment model developed by Thomas and Velthouse (1990) underscores the importance of considering the situational attribute of empowerment, which pertains to individuals’ perceptions of the existing environment (9). An individual’s psychological empowerment is influenced by environmental factors, their interpretive style, and their overall assessment of the situation. This sense of empowerment shapes an individual’s behavior and could potentially alter their surroundings. The model also indicates that environmental factors and individual explanatory styles can interfere with psychological empowerment (9). Spreitzer’s widely recognized psychological empowerment theory builds upon the theoretical model proposed by Thomas (8). A systematic review has confirmed the link between environmental factors and psychological empowerment: structural empowerment (environmental influences) leads to psychological empowerment, resulting in positive workplace outcomes like job satisfaction. As an outcome of structural empowerment, psychological empowerment also helps individuals understand how structured work environments affect key organizational performance outcomes (44). In this context, psychological empowerment acts more as a mediating variable. Overall, any interventions designed to enhance nurses’ psychological empowerment should consider both environmental factors and individual perceptions.

Some studies, such as those employing group psychological counseling and positive mindfulness therapy (28, 29, 40, 41), while related to psychological empowerment or psychotherapy, did not explicitly address support for the nurses’ work environment. Although they consider nurses’ perceptions to some extent, they do not directly address the support needed for their work environment. They focused more on directly intervening in the nurses’ psychological states, without specifically empowering them from an organizational perspective by providing appropriate information, support, resources, and opportunities. In these studies, psychological empowerment is utilized as a metric for mental health-related outcomes. These interventions failed to differentiate themselves from interventions targeting other psychologically related concepts, such as job satisfaction or burnout. Therefore, future research should aim to develop a deeper understanding and application of the core principles of psychological empowerment. This includes considering the situational aspects of empowerment when designing intervention content and approaches. It is crucial to recognize that structural empowerment, which involves organizational support, is a necessary condition for psychological empowerment. Furthermore, there should be an improved analysis and understanding of the context in which nurses operate, with support and resources tailored to more effectively achieve the objectives of psychological empowerment.

The specific descriptions of the interventions indicated that the interventions were comprehensive. The relevant course training and theoretical studies were instrumental in helping nurses understand the significance of psychological empowerment and the appropriate pathways to achieve it. Additionally, the form and duration of the interventions were flexible and lacked standardized theoretical training or the length of applied practice. For instance, LiChun Chang (37) designed a 4-week, 60-min once-weekly empowerment education program, with 61 out of 64 invited participants completing the entire course. In another study involving online learning over 19 months, only 44 out of 85 participants were willing to continue after the program concluded (35). This indicates the need to adjust the intervention’s length flexibly based on the participants’ specific circumstances. Additionally, considering the limitations of offline training in terms of time and geography, it is crucial to diversify the forms of psychological empowerment interventions by actively utilizing Internet platforms. For example, MacPhee and Dahinten (23) established an online knowledge network to enhance the connections among novice frontline nurse leaders in the western provinces of Canada who participated in the program. Terkamo-Moisio and Peltonen (35) collaborated with five universities in Finland to provide an online course for current and future nurse leaders from nine different healthcare organizations. Given the current shortage of clinical nurses and the scarcity of high-quality educational resources, researchers should consider developing online training courses in collaboration with multiple organizations. This approach would enable participants to progress through the course at their own pace, providing them with flexibility and autonomy to maximize the utilization of intervention resources. At the same time, the quality control and evaluation of the effectiveness of the online training course should be enhanced to ensure that it truly meets the needs of nurses and enhances their level of psychological empowerment.

It is interesting to note that the existing studies exhibited a higher level of consistency and less heterogeneity in their selection of psychological empowerment assessment tools. This suggests that intervention effects can be explored based on evidence-based criteria. Furthermore, considering the causal relationship between structural empowerment and psychological empowerment (45), it is recommended that future studies include structural empowerment as an outcome indicator alongside psychological empowerment. In terms of measuring structural empowerment, the included studies predominantly utilized the Laschinger CWEQ-II assessment tool, indicating a commonality in this aspect (46, 47). It is therefore encouraged that future studies incorporate the Spreitzer PEI alongside the Laschinger CWEQ-II for a more comprehensive assessment. However, it is important to recognize that self-report measures can be more susceptible to social desirability bias or response bias. To mitigate this potential issue, future studies should consider using a combination of measures, such as incorporating objective indicators, to improve the accuracy and reliability of the measurements.

The inclusion of point-in-time measurements in the studies was commendable as it allowed for the assessment of both short- and long-term effects of the interventions. Many of the studies included in this review incorporated a follow-up period after the intervention, which is valuable for demonstrating the long-term effects. Nevertheless, it is important to strike a balance when setting measurement time points, as setting too many or too long intervals may result in sample attrition, waste of resources, and increased exposure to other sources of empowerment, potentially leading to data errors. Future studies should consider these factors and design appropriate measurement time points based on specific intervention programs to accurately evaluate their effects. Additionally, researchers should explore how different measurement time points influence the assessment of intervention outcomes and how to optimize these settings to enhance the scientific validity and effectiveness of the study.

Finally, despite the effectiveness of interventions in increasing the level of nurses’ psychological empowerment, some studies included in the review did not demonstrate a significant overall increase in the level of psychological empowerment. Additionally, two studies within the review employed direct intervention with nurse managers to observe changes in the psychological empowerment of both the nurse managers themselves and the nurses under them (referred to as nurse followers). One study showed a significant increase in the psychological empowerment level of the nurse followers, along with significant differences in the impact sub-dimensions (24). However, the other study found a non-significant change in the psychological empowerment of the nurses (23, 39).

These discrepancies may be related to the nurses’ perceptions of their nurse managers’ leadership. One study has indicated that nurses’ perceptions of authentic leadership exhibited by nurse managers can enhance all three dimensions of psychological empowerment: meaning, self-determination, and competence (11). Moreover, factors such as the duration of the intervention, the extent to which the training content is effectively utilized, and individual differences among the participants may also play a role in influencing the results. Future research could study deeper into the specific mechanisms through which these factors influence intervention outcomes, expand the breadth and depth of the study, and propose more targeted intervention strategies to further enhance the effectiveness of psychological empowerment interventions for nurses. At the same time, attention can be paid to the impact of individual differences on the effectiveness of interventions, and personalized interventions can be carried out to meet the needs of different nurses.

## 5 Limitations

(1) The review focused only on articles published in English and Chinese; therefore, some studies in other languages may have been missed. (2) Although the search incorporated the maximum number of keywords known to retrieve research on interventions related to supporting nurses’ psychological empowerment, there may have been relevant keywords that were left out, which could have affected the conclusions. (3) The heterogeneity of intervention effects poses a challenge when synthesizing evidence supporting nurses’ psychological empowerment. This review was conducted at the scoping level, and systematic reviews and correlational meta-analysis are recommended as follow-ups to explore additional research. The growing amount of research on nurses’ psychological empowerment shows that the field is thriving. A systematic review would be useful for keeping up with current evidence and making recommendations for best practices and future research.

## 6 Conclusion

This study has demonstrated the ongoing refinement and enrichment of studies on interventions aimed at supporting the psychological empowerment of nurses. Various strategies, such as the Empowerment-based educational program, Multi-faceted educational program, Nursing Leadership Institute (NLI) program, and Psychoeducational group program, have been implemented, showing promising positive impacts. However, some limitations need to be addressed in future research. It is recommended that future studies employ randomized controlled trials with larger sample sizes. Furthermore, choosing appropriate theoretical frameworks to design intervention programs based on the core concept of psychological empowerment to improve the scientific nature and validity of the research is highly recommended. Meanwhile, the content and form of the intervention should be diversified, and creating more targeted programs that align with the actual needs and working environment of nurses. Additionally, we should reinforce the evaluation of both the implementation process and the effectiveness of these interventions using various methods and indicators to comprehensively and accurately assess their impact.

## Data Availability

The original contributions presented in the study are included in the article/Supplementary material, further inquiries can be directed to the corresponding author.

## Footnotes

^1^ https://opengrey.eu/
