# Supplementary material for: Interventions to support the psychological empowerment of nurses: a scoping review
Source: Front Public Health. 2024 Dec 9;12:1427234. doi: 10.3389/fpubh.2024.1427234 (PMC11664262; doi:10.3389/fpubh.2024.1427234)
Supplement: Supplementary file 1 [file Table_1.DOCX]

**Search strategy 2024**

**（1）PubMed/Medline** **5-9-2024**

| # | Query | Results |
| --- | --- | --- |
| #1 | ((((((((((((("Nurses"[Mesh]) OR (Nurs*[Title/Abstract])) OR (Personnel, Nursing[Title/Abstract])) OR (Nursing Personnel[Title/Abstract])) OR (Registered Nurses[Title/Abstract])) OR (Nurse, Registered[Title/Abstract])) OR (Nurses, Registered[Title/Abstract])) OR (Registered Nurse[Title/Abstract])) OR (nurse practitioner*[Title/Abstract])) OR (staff nurs*[Title/Abstract])) OR (nursing staff*[Title/Abstract])) OR (nurse clinician*[Title/Abstract])) OR (licensed practical nurs*[Title/Abstract])) OR (nurse midwi*[Title/Abstract]) | 596552 |
| #2 | (Psychological Empowerment[Title/Abstract]) OR (Psychological-Empowerment[Title/Abstract]) | 506 |
| #3 | ((((((((randomized controlled trial[Publication Type]) OR (controlled clinical trial[Publication Type])) OR (randomized[Title/Abstract])) OR (randomly[Title/Abstract])) OR (trial[Title/Abstract])) OR (groups[Title/Abstract])) OR (quasi-experimental[Title/Abstract])) OR (before[Title/Abstract] AND after study[Title/Abstract])) OR (controlled before-after study[Title/Abstract]) | 3976806 |
| #4 | #1 AND #2 AND #3 | 30 |

**（2）Web of Science 5-9-2024**

| # | Query | Results |
| --- | --- | --- |
| #1 | TS=(Nurs*) OR TS=(Personnel, Nursing) OR TS=(Nursing Personnel) OR TS=(Registered Nurses) OR TS=(Nurse, Registered) OR TS=(Nurses, Registered) OR TS=(Registered Nurse) OR TS=(nurse practitioner*) OR TS=(staff nurs*) OR TS=(nursing staff*) OR TS=(nurse clinician*) OR TS=(licensed practical nurs*) OR TS=( nurse midwi*) | 1430919 |
| #2 | TS=(Psychological Empowerment) OR TS=( Psychological-Empowerment) | 12616 |
| #3 | ((((((((TS=(randomized controlled trial)) OR TS=(controlled clinical trial)) OR TS=(randomized)) OR TS=(randomly)) OR TS=(trial)) OR TS=(groups)) OR TS=(quasi-experimental)) OR TS=(before and after study)) OR TS=(controlled before-after study) | 17471911 |
| #4 | #1 AND #2 AND #3 | 674 |

**（3）Scopus 5-9-2024**

| # | Query | Results |
| --- | --- | --- |
| #1 | ( TITLE-ABS-KEY ( nurs* ) OR TITLE-ABS-KEY ( nursing AND personnel ) OR TITLE-ABS-KEY ( registered AND nurses ) OR TITLE-ABS-KEY ( registered AND nurse ) OR TITLE-ABS-KEY ( nurse AND practitioner* ) OR TITLE-ABS-KEY ( staff AND nurs* ) OR TITLE-ABS-KEY ( nursing AND staff* ) OR TITLE-ABS-KEY ( nurse AND clinician* ) OR TITLE-ABS-KEY ( licensed AND practical AND nurs* ) OR TITLE-ABS-KEY ( nurse AND midwi* ) ) | 1031790 |
| #2 | TITLE-ABS-KEY ( psychological-empowerment ) | 2035 |
| #3 | ( TITLE-ABS-KEY ( randomized AND controlled AND trial ) OR TITLE-ABS-KEY ( controlled AND clinical AND trial ) OR TITLE-ABS-KEY ( randomized ) OR TITLE-ABS-KEY ( randomly ) OR TITLE-ABS-KEY ( trial ) OR TITLE-ABS-KEY ( groups ) OR TITLE-ABS-KEY ( quasi-experimental ) OR TITLE-ABS-KEY ( before AND after AND study ) OR TITLE-ABS-KEY ( controlled AND before-after AND study ) ) | 16389487 |
| #4 | #1 AND #2 AND #3 | 81 |

**（4）Embase 5-9-2024**

| # | Query | Results |
| --- | --- | --- |
| #1 | nurs* OR 'personnel, nursing':ab,ti OR 'nursing personnel':ab,ti OR 'registered nurses':ab,ti OR 'nurse, registered':ab,ti OR 'nurses, registered':ab,ti OR 'registered nurse':ab,ti OR 'nurse practitioner*':ab,ti OR 'staff nurs*':ab,ti OR 'nursing staff*':ab,ti OR 'nurse clinician*':ab,ti OR 'licensed practical nurs*':ab,ti OR 'nurse midwi*':ab,ti | 3900168 |
| #2 | 'psychological empowerment' | 453 |
| #3 | 'randomized controlled trial'/exp OR 'randomized controlled trial' OR (randomized AND controlled AND ('trial'/exp OR trial)) OR 'controlled clinical trial':ab,ti OR randomized:ab,ti OR randomly:ab,ti OR trial:ab,ti OR groups:ab,ti OR 'quasi experimental':ab,ti OR (before:ab,ti AND 'after study':ab,ti) OR 'controlled before-after study':ab,ti | 5579313 |
| #4 | #1 AND #2 AND #3 | 41 |

**（5）****EBSCOhost 5-9-2024**

| # | Query | Results |
| --- | --- | --- |
| #1 | SU nurs* OR SU personnel, nursing OR SU nursing personnel OR SU registered nurses OR SU nurse, registered OR SU nurses, registered OR SU registered nurse OR SU nurse practitioner* OR SU staff nurs* OR SU nursing staff* OR SU nurse clinician* OR SU licensed practical nurs* | 2564295 |
| #2 | TI psychological empowerment OR SU psychological empowerment OR AB psychological empowerment | 5275 |
| #3 | SU randomized controlled trial OR SU controlled clinical trial OR SU randomized OR SU randomly OR SU trial OR SU groups OR SU quasi-experimental OR SU ( before and after study ) OR SU controlled before-after study | 4385537 |
| #4 | #1 AND #2 AND #3 | 4 |

**（6）****Cochrane Library 5-9-2024**

| # | Query | Results |
| --- | --- | --- |
| #1 | MeSH descriptor: [Nurses] explode all trees | 1749 |
| #2 | (nurs*):ti,ab,kw OR (personnel, nursing):ti,ab,kw OR (nursing personnel):ti,ab,kw OR (registered nurses):ti,ab,kw OR (nurse, registered):ti,ab,kw | 59660 |
| #3 | (nurse practitioner*):ti,ab,kw OR (staff nurs*):ti,ab,kw OR (nursing staff*):ti,ab,kw OR (nurse clinician*):ti,ab,kw OR (licensed practical nurs*):ti,ab,kw | 11486 |
| #4 | (psychological-empowerment):ti,ab,kw (Word variations have been searched) | 54 |
| #5 | (randomized controlled trial):ti,ab,kw OR (controlled clinical trial):ti,ab,kw OR (randomized):ti,ab,kw OR (randomly):ti,ab,kw OR (ltrial):ti,ab,kw OR (groups):ti,ab,kw OR (quasi-experimental):ti,ab,kw OR (before and after study):ti,ab,kw OR (controlled before-after study):ti,ab,kw | 1507976 |
| #6 | #1 or #2 or #3 | 59660 |
| #7 | #6 and #5 and #4 | 18 |

**（7）****OpenGrey 5-9-2024**

**<https://easy.dans.knaw.nl/ui/datasets/id/easy-dataset:317654>**  1

**（8）CNKI 5-9-2024**

| # | Query | Results |
| --- | --- | --- |
| #1 | (篇关摘=心理授权) | 744 |
| #2 | (篇关摘=护士) OR (篇关摘=护理人员) OR (篇关摘=临床护士) | 243600 |
| #3 | (篇关摘=随机对照试验) OR (篇关摘=随机) OR (篇关摘=对照) OR (篇关摘=RCT) OR (篇关摘=干预前后) OR (篇关摘=干预) OR (篇关摘=前后) OR (篇关摘=试验) | 6674100 |
| #4 | #1 AND #2 AND #3 | 37 |

**（9）****Wanfang 5-9-2024**

| # | Query | Results |
| --- | --- | --- |
| #1 | 主题:(心理授权) | 1298 |
| #2 | 主题:(护士) or 主题:(护理人员) or 主题:(临床护士) | 291336 |
| #3 | 主题:(随机对照试验) or 主题:(随机) or 主题:(对照) or 主题:(干预) or 主题:(干预前后) or 主题:(前后) | 5167193 |
| #4 | #1 AND #2 AND #3 | 42 |

**（10）VIP 5-9-2024**

| # | Query | Results |
| --- | --- | --- |
| #1 | 主题=心理授权 | 2957 |
| #2 | 主题=护士 OR 主题=护理人员 OR 主题=临床护士 | 402100 |
| #3 | 主题=随机对照试验 OR 主题=随机 OR 主题=对照 OR 主题=干预 OR 主题=干预前后 | 7214200 |
| #4 | #1 AND #2 AND #3 | 89 |
